# Ash Dieback and Its Impact in Near-Natural Forest Remnants – A Plant Community-Based Inventory

**DOI:** 10.3389/fpls.2019.00658

**Published:** 2019-05-24

**Authors:** Alexandra Erfmeier, Kerstin L. Haldan, Lili-M. Beckmann, Magdalene Behrens, Jonas Rotert, Joachim Schrautzer

**Affiliations:** ^1^Institute for Ecosystem Research, Geobotany, Kiel University, Kiel, Germany; ^2^German Centre for Integrative Biodiversity Research (iDiv) Halle-Jena-Leipzig, Leipzig, Germany; ^3^Applied Ecology, Institute for Ecosystem Research, Kiel University, Kiel, Germany

**Keywords:** *Hymenoscyphus fraxineus*, fungal pathogen, *Fraxinus excelsior*, ash rejuvenation, forest community type, ash-rich forests, species richness, ecosystem vulnerability

## Abstract

Temperate European forests are currently largely under attack by the infection with *Hymenoscyphus fraxineus*, a fungal pathogen introduced from Asia since at least the early 1990s and causing a major dieback of common ash (*Fraxinus excelsior*) throughout Europe. At present, ash dieback evokes major problems for forestry, in particular in sensitive forest remnants in Northern Germany, where the disease causes serious concerns for ecosystem conservation. This makes ash dieback a focal area of ecological research. In the present study, we quantified the extent of ash dieback in adult and in young ash trees in Northern Schleswig-Holstein, Germany, in relation to community composition and associated biotic and abiotic factors. Data collection was carried out in 37 plots in communities of ash-rich forests and included floristic inventory, rating of adult and young ash individuals and recording of light and soil conditions. Data were analyzed using non-metric multidimensional scaling and general linear mixed effects models. Forest type was the strongest significant predictor for variation in crown defoliation of adult ash trees. Damage was highest in communities of wet alder-ash forests and lowest in ash-rich beech forests. A further significant predictor of adult crown defoliation was individual height of the ash tree with larger trees being less affected than smaller ones. For juveniles, total species richness displayed a significant positive relationship with the proportional abundance of fungal infection, while the mean damage proportion per individual significantly increased with increasing relative light intensity in the understorey. The study clearly shows a strong relationship between forest type and ecosystem vulnerability to ash dieback. In particular, communities belonging to the species-rich wet alder-ash forests were most severely affected by ash disease, thereby deserving special attention among the vulnerable fragmented forest remnants in Schleswig-Holstein. Co-varying factors, however, seem to differ between juvenile and adult trees, hinting at the relative importance of tree performance for the adult trees and abiotic conditions for the juveniles. Accounting for such differences along a larger ecological gradient of ash forest communities will be necessary to more comprehensively understand effects of ash dieback on the ecosystem and needs to be addressed in future research.

## Introduction

Due to the globalization of human activities such as world-wide trade of goods, species are translocated across the globe (Mack et al., 2000) and introduced into areas they would otherwise not be able to colonize (Richardson et al., 2000). Some of these species become invasive with the detrimental potential to alter whole ecosystems (Mack et al., 2000; Richardson et al., 2000; Bradley et al., 2010). In the case of fungal pathogens, large-scale infectious diseases can develop even more complex multitrophic interactions and, thus, may affect ecosystem functioning. Such emerging infectious diseases (Jönsson and Thor, 2012) are currently of great concern. At present, the ash dieback is a fungal infection disease, largely affecting common ash (*Fraxinus excelsior* L.) throughout its native range in temperate Europe and causing a severe dieback of this species (Kowalski et al., 2010; Kowalski et al., 2015; Dietrich, 2016). It is expected that a high proportion of ashes will die off and threaten the survival of *F. excelsior* in its natural habitats in the near future (Pautasso et al., 2013). The loss of ashes will evoke major economic and ecological consequences: Common ash produces high-quality timber (Pautasso et al., 2013; Rutjes, 2017) and in European forest ecosystems, the species fulfills ecological functions (Pautasso et al., 2013; Fussi and Konnert, 2014) as it is an important pioneer and gap-filling species (Mitchell et al., 2014; Rutjes, 2017) producing leaf litter that is easily decomposable and enabling rapid nutrient cycling in the forest (Wardle, 1961; Jacob et al., 2010). Ash foliage permits high amounts of light to penetrate the overstorey, creating a favorable light climate for understorey vegetation, thereby influencing diversity of herb and shrub layer community composition (Emborg, 1998; Härdtle et al., 2003).

The ash dieback is caused by infection with *Hymenoscyphus fraxineus* (T. Kowalski) Baral, Queloz, Hosoya (Baral et al., 2014; Kowalski et al., 2015; Lenz and Straßer, 2016), an ascomycete native to East Asia and introduced to Poland in the 1990s (Östbrant et al., 2017). Since then, it has spread all over Europe (Chavez et al., 2015), reaching Northern Germany presumably in 2002, and now being present in a large part of the distribution area of *F. excelsior* (Kowalski et al., 2010, 2015; Dietrich, 2016). The ash dieback threatens not only *F. excelsior* as its host but also much of the biodiversity associated with it. Although it is well known that such extensive diseases diminishing species occurrence can have a strong impact on the ecosystem (Loo, 2009; Jönsson and Thor, 2012), their feedback on the ecosystem has not been studied thoroughly in the past (Loo, 2009).

General knowledge, to date, suggests several factors to be involved in explaining the vulnerability to the disease. On the one hand, the extent of infection damage has been reported to vary with environmental conditions and properties of the infested trees. While generally tree individuals can be affected independent of age and size, and infections may occur in many habitat types, some studies report a variation in the severity of the disease (Kowalski and Holdenrieder, 2008; Pautasso et al., 2013; Lenz and Straßer, 2016). For example, there is evidence that ashes at wet sites may suffer from more severe damage from ash dieback than at moist and base-rich sites (Kenigsvalde et al., 2010; Schumacher, 2011; Lenz and Straßer, 2016). Yet, on the other hand, biotic covariation, as induced either by the performance of the tree species themselves, community characteristics associated with diversity and/or forest management may affect responses of *F. excelsior* individuals to the infection. For example, Bakys et al. (2013) reported a relation to ash stand density, and they found effects of ash dieback to be stronger in dense, unthinned plots compared to managed, thinned plots. In addition, age and size of a tree can be expected to have an influence on the course of an infection. A study in Latvia, e.g., revealed a difference of the extent of ash dieback between age groups, with young stands showing higher rates of decline than older ones (Kenigsvalde et al., 2010). Generally, smaller and less vital trees are presumed to be more susceptible to the disease while old trees die off slower (Skovsgaard et al., 2010; Thomas, 2016).

To date, there is a mosaic of information available on the mechanisms of fungal infection, development of the disease (Thomas, 2016) and also data on mortality rates are reported either in an European-wide census and meta-analysis (Coker et al., 2019) or in forest stand-based observations for single regions, e.g., in Latvia, Sweden or Norway (Kenigsvalde et al., 2010; Bengtsson et al., 2013; Timmermann et al., 2017). However, a thorough analysis of ash dieback extent in the context of a more comprehensive assessment of ecosystem variation in ash-rich forests is ultimately missing.

In Northern Germany, forests, by the majority, occur as postglacially imprinted remnants superimposed by management efforts for centuries, and the proportion of forest cover is largely underrepresented in comparison to other regions in temperate Europe (Häme et al., 2001). Therefore, ecological research in order to capture the extent and the context-dependency of forest susceptibility is highly demanded also for reasons of conservation. Northern Germany, and in particular the federal state of Schleswig-Holstein therein is exceptionally well-suited for such an approach since the region offers site conditions representing almost the whole abiotic gradient of common ash in Central Europe. Moreover, and due to the early occurrence of infestation, the progression of the disease can be studied more profoundly in this region than in more southern parts of Europe.

In the present study, we quantified the extent of ash dieback in adult and in young ash trees at 37 sites located in the northern part of Schleswig-Holstein in order to contribute to filling the research gaps and to test the following hypotheses: (1) Infection damage of adult ash trees differs between different forest types and landscape types, and we expect an increasing infection from dry to wet forest community types, (2) damage patterns of juvenile ashes vary accordingly along the gradient of forest type communities, and (3) a significant proportion of variation in damage patterns can be assigned to covariation with biotic and abiotic variables.

## Materials and Methods

### Study System

Common ash (*F. excelsior* L.) is a deciduous tree species native to Europe and widespread throughout its range. Depending on the site conditions, adult trees can reach a size in height from 10 to 40 m (Jäger and Rothmaler, 2017) with a diameter at breast height of 1.6–1.8 m (Thomas, 2016). Ashes usually grow in mixed stands in deciduous forests covering a considerable range of soil conditions, ranging from (dry-) mesic to wet stands (Wardle, 1961), including mineral to organic soils with subacidic to alkaline ground reactions (Thomas, 2016). Leaves are characterized by having a high nutrient and a low lignin content indicating a high nutrient availability of sites. Thus, ash leaf litter is easily decomposable and nutrients are rapidly being retransferred into forest nutrient cycling (Wardle, 1961; Jacob et al., 2010; Langenbruch et al., 2012). In closed forest stands, *F. excelsior* has a narrow crown that fosters light demanding species in the understorey (Härdtle et al., 2003). While ash seedlings and juveniles are shade-tolerant, they become more light-demanding when reaching 0.5 m in height (Marigo et al., 2000).

*Hymenoscyphus fraxineus* is an ascomycete native to East Asia (Zhao et al., 2012; Zheng and Zhuang, 2014; Cleary et al., 2016; Thomas, 2016), where it colonizes congeneric *F. mandshurica* Rupr. and *F. chinensis* Roxb. as host species, mostly not causing any harm to the trees (Zhao et al., 2012; Gross and Han, 2015; Gross and Holdenrieder, 2015; Cleary et al., 2016). For Schleswig-Holstein, *H. fraxineus* was first referenced in 2002 and laboratory-confirmed in 2005 (Langer, 2017). The ascomycete forms fruiting bodies on ash leaf litter, releasing ascospores in the summer months (Lenz and Straßer, 2016). The infection process starts with windborne spores infecting ash leaves. The fungus enters a leaf via the rhachis, hyphae grow into the pith of the twigs and branches and later proceed into the lignified areas of the tree.

The first visible symptom of the disease is the brown discoloration of infected leaves (Kowalski and Holdenrieder, 2008; Lenz and Straßer, 2016; Thomas, 2016). In the course of the disease, bark necroses occur (Schumacher et al., 2007; Kowalski and Holdenrieder, 2008; Lenz and Straßer, 2016) as the cambium and external tissues die off (Thomas, 2016). Necroses girdling the trunk interrupt the water flow and finally kill the tree (Kowalski et al., 2010; Lenz and Straßer, 2016; Thomas, 2016). Young ashes are reported to die off faster than old trees (Schumacher, 2011). While juvenile ashes usually die off in the course of months, older trees often show a chronic course of the disease (Kowalski et al., 2010) where trees develop crown dieback and bastard branches leading to a bushy growth (Schumacher et al., 2007; Kowalski and Holdenrieder, 2008; Kowalski et al., 2010).

### Study Area

**FIGURE 1:**
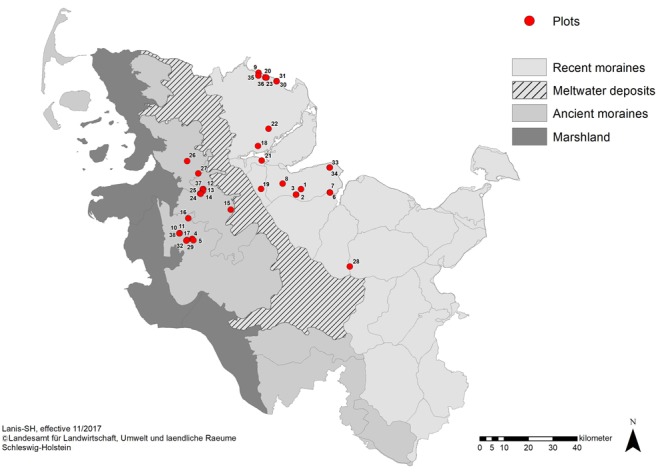
Main landscape regions of Schleswig-Holstein with locations of forest plots. The two investigated landscape types are the ancient moraines in the west (gray) and recent moraines in the east (light gray). Location of forest plots is depicted by red dots.

Research was carried out in the northern part of Schleswig-Holstein, Germany (Figure 1). In the study area, the mean annual temperature is 8.9°C (1998–2010) and the mean annual precipitation is 823 mm (1981–2010) (DWD, 2017). The landscape in Schleswig-Holstein is largely shaped by its glacial and postglacial formation history: From the East to the West, forests in Schleswig-Holstein occur in the landscape regions of recent moraines (“*Östliches Hügelland*”) and ancient moraines (“*Hohe Geest*”), which differ in landscape, soil type, and forest vegetation characteristics (Gripp, 1964). Recent moraines originated in the last (Weichselian) glaciation and are characterized by a hilly landscape form. Postglacial soil development gave rise to luvic Arenosols/orthic Luvisols that can turn into gleyic Luvisols at high levels of soil moisture with a potential natural vegetation of beech (*Fagus sylvatica* L.) forests. The wet extreme of the soil water gradient is represented by Gleysols with alder-ash-riparian forests (*Alnus-Fraxinus* forests) under the influence of ground water (Härdtle et al., 2003).

The ancient moraines were formed during the penultimate (Saale) glaciation. On ancient moraines, Podzols predominate with beech-oak forests (*Fagus-Quercus* forests) (LLUR, 2012). The recent forest landscape in Schleswig-Holstein is largely fragmented and represents a mosaic of ancient forest remnants and reforested, managed stands, usually distributed in small patches. Amounting to 173,412 hectares, forest covers only 11 % of the total area, making Schleswig-Holstein the federal state with least forest area in Germany (Bundesministerium für Ernährung und Landwirtschaft [BMEL], 2016).

### Plot Sampling Design

In the present study, sampling addressed forest stands that represent almost the whole moisture gradient of ash on mineral soils from dry to wet conditions, not including alluvial forests and drained sites with organic soils. The following three forest vegetation types were selected to be implemented in the study:

(1) Alder-ash forests (AAF) as part of the alliance Alno-Ulmion, i.e., wet forests with black alder [*Alnus glutinosa* (L.) GäRTN.] and common ash (*F. excelsior*) growing on gleysols,
(2) Hornbeam-ash forests (HAF) as part of the alliance Alno-Ulmion, i.e., moderately wet forests with black alder and a mixture of several other deciduous trees including the European hornbeam (*Carpinus betulus* L.) growing on stagnosols,
(3) Beech-ash forest (BAF) as part of the alliance Fagion sylvaticae, i.e., moist base-rich forests with European beech (*Fagus sylvatica* L.) and common ash growing on luvisols.

Beech forests with ash admixtures (BAF) represent the driest conditions on soils with a large supply of nutrients and bases, however, with enough moisture as they are poorly drained (Ellenberg and Leuschner, 2010). Species richness in beech-ash forests is usually lower than in the other two forest types addressed in this study. Alder-ash forests (AAF) represent the wettest forest type considered in this study and are characterized by year-round soil wetness and usually good nutrient supply. Upper soil layers are rich in humus, and alder and ash are typically the dominant tree species. Alder-ash forests belong to the most species-rich vegetation types in the Northern German lowlands. Hornbeam-ash forests having a higher soil moisture than beech forests but drier soils than the alder-ash forests represent a transitional forest type – intermediate in site conditions and species richness – characterized by a high base saturation and a narrow C/N-ratio. Here, *F. excelsior* is usually the dominating tree species under natural competitive conditions (Ellenberg and Leuschner, 2010).

We applied a stratified and balanced sampling design across the three forest types in order to represent all typical forest community types appropriately in the present study. Between May and August 2017, 37 study plots were established in 23 ash-rich forest areas in Schleswig-Holstein. The plots were randomly selected evenly filling the three forest types each in the two landscape types: the final selection included 18 and 19 plots of the landscape types ancient moraines and recent moraines, respectively (Figure 1), and representing at least six plots each in the three forest types ash-alder forests (AAF), hornbeam-ash forests (HAF) and beech forests (BAF, with six plots in ancient moraine landscape and seven plots in recent moraine landscape, respectively).

### Data Collection

Each plot had a size of 10 × 10 m and was oriented toward the north. Within each plot, composition and cover of all vascular plant species and mosses were recorded according to the LONDO scale (Londo, 1976). Nomenclature of vascular plants and mosses followed Jäger and Rothmaler (2017) and Frahm and Frey (1992), respectively.

Species inventory was assessed separately by forest strata: Cover of the upper tree layer (i.e., trees higher than 10 m, TL1), lower tree layer (i.e., trees of 4–10 m height, TL2), shrub layer (i.e., woody species of 1–4 m height, SL), herb layer (i.e., herbaceous species up to 1 m height, HL), mosses (excluding lignicole mosses, M).

Proportion of ashes in the upper tree layer was calculated in relation to total tree layer cover.

### Rating of *Fraxinus excelsior* Individuals

Effects of ash dieback were quantified on the plot level and on the individual level. Abundance of adult ash trees per plot was counted, adult ashes being defined as ash trees with a diameter at breast height (DBH) of at least 15 cm and a height of at least 4 m. On the individual level, each of five adult ashes per plot were systematically selected for additional rating: the individual closest to each one of the four corner points and the one to the center of the plot were selected, respectively. For each selected individual, basal stem diameter, DBH and total height were recorded. Damage due to the ash dieback was recorded as percentage of crown defoliation and in six damage classes following Lenz et al. (2012). In addition, we quantified wood necroses on ash trunks in three classes. As this variable was highly significantly correlated with leaf damage (Spearman’ rho stat: *r* = 0.902, *p* < 0.001, data not shown), necrosis was not further considered in the analyses.

To assess the amount of ash rejuvenation in a plot, all juvenile ashes up to 4 m in height were counted, excluding seedlings with cotyledons only. Damage induced by *H. fraxineus* was assessed for each juvenile ash individual as presence/absence record of infection and quantified as relative damage by individual in percent, later calculating proportion of damaged juveniles by plot.

### Environmental Site Conditions

Photosynthetically active radiation (PAR) was measured as photon flux density (PFD, mmol/m^2^s^-1^, wavelength 400–700 nm) with the SunScan Canopy Analysis System (Delta T Devices Ltd., Cambridge, United Kingdom). Light measurements were carried out during full foliation of the forest. Plots were subdivided into nine equal subplots and two measurements were carried out above the herb layer at a standardized measuring height of 1.3 m in each of the subplots. Relative light intensity (RLI) was calculated per plot as a mean across all subplots.

Soil samples for determination of pH, CN, and cation exchange capacity (CEC) were taken from a central location in each plot, at a depth of 10 cm. Soil samples were sieved and air dried. PH was measured potentiometrically in a 1:2.5 soil: H_2_O and 0.01 molar CaCl_2_ solution using the pH-meter Lab 860 Sen Tix HW (Schott Instruments). Total carbon and nitrogen were determined on dried (24 h at 105°C) and ground soil samples using the C/N-Analyzer EURO EA 3000 (HEKAtech GmbH, Wegberg, Germany). Since all soil samples are non-calcareous, the measured total C content equals organic carbon (C_org_).

Extraction of exchangeable cations was performed on 2.5 g of air dried soil with 0.1 molar BaCl_2_. The concentrations of the exchangeable cations Na^+^, K^+^, Ca^2+^, Mg^2+^, Al^3+^, Fe^3+^, and Mn^2+^ were measured using atomic absorption spectrophotometry (AAS), and CEC and base saturation (BS) were calculated.

### Statistical Analysis

Floristic composition of all species was analyzed with non-metric multidimensional scaling (NMDS) using Bray-Curtis dissimilarity. Environmental variables were ex post-fitted to the ordination graph. Significance of correlation with NMDS axes was tested with a permutation test (*n* = 999). NMDS was performed using the vegan package (Oksanen et al., 2018).

The relationship between ash dieback and environmental variables was explored further using linear mixed-effect models (LMM). Before running the models, data were checked for outliers, zero inflation and autocorrelation among predictor variables. In case of significant correlation (significance threshold r_Spearman_ = 0.7) only one of the variables was included in the model. Residual plots of each selected model were examined to ensure random distribution of model residuals.

A first model was run on the plot level incorporating all environmental information that was only available as one sample per plot. In a full model, we tested for effects of landscape type and forest type, and their interaction as fixed factors explaining mean crown defoliation of adult ash trees. In addition, mean diameter at breast height and mean height of adult ashes, the abundance of adult ashes and their proportion in the upper tree layer (TL1), the abundance of juveniles, and total species richness were included as biotic covariates just as soil pH and soil CN as abiotic covariates. Forest area ID was considered a random factor. Prior to analysis, each numerical predictor variable was centered around its mean. Final models were selected by comparing all possible predictor combinations based on Akaike Information Criterion with correction for small sample sizes (AICc; Sugiura, 1978). Models were fitted to the data via maximum likelihood (ML), the final model was fitted using restricted maximum likelihood (REML). Significant differences between factor levels were tested with *post hoc* pairwise *t*-tests.

A second model was run on the individual level testing for effects of environmental variables on loss of leaves of the individual adult ash trees. Again, landscape type, forest type, and their interaction were considered fixed effects. We included height and DBH of individual ash trees as covariates in the model and considered forest area ID and plot ID nested in forest area ID random effects. The model was fitted to the data via REML. Significant differences between factor levels were tested with *post hoc* pairwise Wilcoxon rank sum tests.

In the third approach, fungal infection damage of juvenile ashes was examined on the plot level. We considered fungal infection as mean response on the individual presence/absence level (i.e., damage was assessed as abundance of occurrence at the individual level) and, in addition, as a mean response of the estimated extent of damage on the individual level (i.e., as the mean of percental damage across all juveniles per plot). Mean percental damage of juveniles was log transformed prior to analysis. In the full model, we tested for effects of landscape type and forest type and their interaction as fixed factors. The plot level environmental variables mean crown defoliation of adult ashes, DBH of adult ashes, mean height of adult ashes, abundance of juvenile ashes, abundance of adult ashes, proportion of ashes in upper tree layer, soil C/N, soil pH, mean understorey RLI, and total species richness were included as covariates. Forest area ID was considered a random effect. Before running the model, each numerical predictor variable was centered around its mean. A set of linear mixed effects models with all possible combinations and subsets of fixed effects was created for the two response variables, each. Models were fitted to the data via maximum likelihood (ML) and ranked according to the second-order Akaike Information Criterion (AIC). The models with lowest AIC value were selected and fitted using REML, respectively.

All statistical analysis were performed in R, version 3.4.0 (R Core Team, 2017). All models were fitted using the lme4 package (Bates et al., 2015). Model selection was performed using the MuMin-package (Bartoń, 2018).

## Results

### Species Composition and Environmental Variables (NMDS)

**FIGURE 2:**
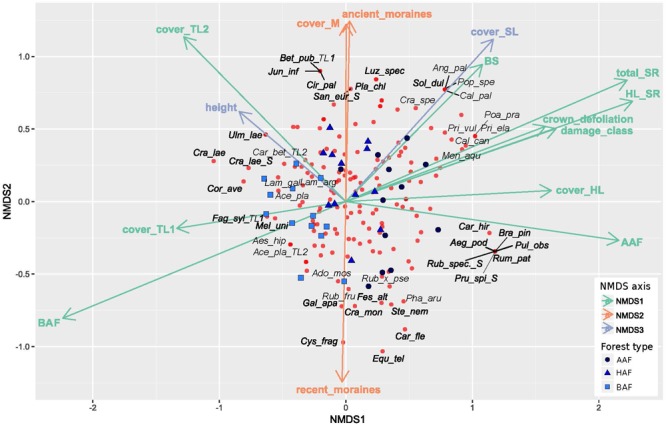
Non-metric multidimensional scaling of cover of all herb layer species in the plots (*n* = 37), with blue symbols indicating the different forest types: alder-ash forest (AAF, circles), hornbeam-ash forest (HAF, triangles) and beech-ash forest (BAF; squares). Species are represented by red circles. Environmental variables that correlate significantly (*p* ≤ 0.05) with one of the axes of the NMDS are shown and colored accordingly (green for correlation with NMDS axis 1, red for correlation with NMDS axis 2, blue for correlation with NMDS axis 3). For a clearer display, only 22% of species are labeled. For abbreviations of predictor variables see Table 1. Species names are abbreviated as follows: Ace_pla: *Acer pseudoplatanus* L., Ado_mos: *Adoxa moschatellina* L., Aeg_pod: *Aegopodium podagraria* L., Aes_hip: *Aesculus hippocastanum* L., Ang_pal: *Angelica palustris* (BESSER) HOFFM., Bet_pub: *Betula pubescens* EHRH., Bra_pin: *Brachypodium pinnatum* (L.) P. BEAUV., Cal_can: *Calamagrostis canescens* (F. H. WIGG.) ROTH, Cal_pal: *Caltha palustris* L., Car_bet: *Carpinus betulus* L., Car_fle: *Cardamine flexuosa* WITH., Car_hir: *Carex hirta* L., Cir_pal: *Cirsium_palustre* (L.) SCOP., Cor_ave: *Corylus avellana* L., Cra_lae: *Crataegus laevigata* (POIR.) DC., Cra_mon: *Crataegus monogyna* JACQ., Cra_spe: *Crataegus spec*., Cys_fra: *Cystopteris fragilis* (L.) BERNH. s. str., Equ_tel: *Equisetum telmateia* EHRH., Fag_syl: *Fagus sylvatica* L., Fes_alt: *Festuca altissima* ALL., Gal_apa: *Galium aparine* L., Jun_inf: *Juncus inflexus* L., Lam_arg: *Lamium argentatum* (SMEJKAL) G. H. LOOS (=*Galeobdolon argentatum* SMEJKAL), Lam_gal: *Lamium galeobdolon* (L.) L. subsp. *galeobdolon* (=*Galeobdolon luteum* HUDS.), Luz_spec: *Luzula spec*., Mel_uni: *Melica uniflora* RETZ., Men_aqu: *Mentha aquatica* L., Pha_aru: *Phalaris arundinacea* L., Pla_chl: *Platanthera chlorantha* CUSTER ex RCHB., Poa_pra: *Poa pratensis* L., Pop_spe: *Populus spec.*, Pri_ela: *Primula elatior* (L.) HILL, Pri_vul: *Primula vulgaris* HUDS., Pru_spi: *Prunus spinosa* L., Pul_obs: *Pulmonaria obscura* DUMORT., Rub_fru: *Rubus fruticosus* agg., Rub_spec.: *Rubus spec*., Rub_×_pse: *Rubus* × *pseudidaeus* (WEIHE) LEJ., Rum_pat: *Rumex patientia* L., San_eur: *Sanicula europaea* L., Sol_dul: *Solanum dulcamara* L., Ste_nem: *Stellaria nemorum* L. s. str., Ulm_lae: *Ulmus laevis* PALL.

The NMDS analysis indicates differences in floristic composition among the three forest types alder-ash forest, hornbeam-ash forest and beech-ash forest (Figure 2 and Table 1). The plots are scattered along NMDS axis 1 and 2 according to their forest type with plots of the beech-ash forest (BAF, squares) displaying significant negative correlation with NMDS axis 1, thereby clustering on the left-hand side of the ordination space, whereas plots of the alder-ash forest (AAF, circles) display a positive correlation with NMDS axis 1 and are located toward the right of the axis (Figure 2 and Table 1). Plots of the hornbeam-ash forest (HAF, triangles) are located in between the two other groups (Figure 2).

**Table 1:** Correlation coefficients of environmental variables and of variables describing species diversity and community structure with the first three axes of the NMDS.

	NMDS1	NMDS2	NMDS3	*r^2^*	*P*	SigCode
**Alder-ash forest (AAF)**	**0.989**	**–0.123**	**–0.085**	**0.529**	**0.001**	**^∗∗∗^**
Hornbeam-ash forest (HAF)	0.138	0.987	–0.083	0.107	0.3	
**Beech-ash forest (BAF)**	**–0.935**	**–0.336**	**0.112**	**0.637**	**0.001**	**^∗∗∗^**
**Ancient moraines**	**0.017**	**0.724**	**–0.689**	**0.328**	**0.002**	**^∗∗^**
**Recent moraines**	**–0.017**	**–0.724**	**0.689**	**0.328**	**0.002**	**^∗∗^**
**Crown defoliation**	**0.885**	**0.291**	**–0.363**	**0.353**	**0.002**	**^∗∗^**
**Damage class**	**0.907**	**0.275**	**–0.318**	**0.373**	**0.002**	**^∗∗^**
Fungal damage to juveniles	0.808	–0.187	–0.559	0.104	0.321	
Basal diameter	–0.159	–0.196	0.968	0.098	0.315	
Diameter at breast height (DBH)	0.004	–0.192	0.981	0.126	0.197	
**Height**	**–0.583**	**0.429**	**0.69**	**0.233**	**0.046**	**^∗^**
Abundance of adult ashes	–0.805	0.569	–0.17	0.199	0.051	.
Total abundance ashes	–0.88	0.322	–0.349	0.136	0.134	
Density of adult ashes	–0.663	0.605	–0.441	0.064	0.494	
Proportion of ashes in tree layer 1 (TL1)	–0.721	0.515	0.464	0.15	0.126	
Abundance of juvenile ashes	–0.881	0.321	–0.348	0.135	0.143	
**Cover tree layer 1 (TL1)**	**–0.849**	**–0.117**	**0.516**	**0.276**	**0.016**	**^∗^**
**Cover tree layer 2 (TL2)**	**–0.748**	**0.662**	**0.048**	**0.327**	**0.009**	**^∗∗^**
**Cover herb layer (HL)**	**0.962**	**0.045**	**–0.268**	**0.316**	**0.004**	**^∗∗^**
**Cover shrub layer (SL)**	**0.578**	**0.555**	**0.598**	**0.452**	**0.001**	**^∗∗∗^**
**Cover mosses (M)**	**0.001**	**0.73**	**0.684**	**0.312**	**0.001**	**^∗∗∗^**
**Total species richness (SR)**	**0.935**	**0.352**	**0.052**	**0.629**	**0.001**	**^∗∗∗^**
**Herb layer species richness (HL SR)**	**0.955**	**0.291**	**0.059**	**0.627**	**0.001**	**^∗∗∗^**
pH	0.663	0.41	0.626	0.166	0.092	.
C/N	–0.848	–0.495	–0.192	0.137	0.141	
Cation exchange capacity (CEC)	0.308	0.214	0.927	0.16	0.095	.
**Base saturation (BS)**	**0.752**	**0.658**	**0.045**	**0.229**	**0.023**	**^∗^**
Understorey relative light (RLI)	0.975	0.186	0.125	0.185	0.087	.

*Variables in bold show significant correlations. SigCode, significance code (0 “^∗∗∗^” 0.001 “^∗∗^” 0.01 “^∗^” 0.05 “.” 0.1 “ ” 1). Letters in brackets give abbreviations used in Figure 2.*

*Post hoc* correlation of NMDS scores with biotic and abiotic covariates indicate significant correlations (Table 1): NMDS axis 1 was significantly positively correlated with total species richness, herb layer species richness, herb layer cover, mean crown defoliation of adult ashes, and mean damage class of adult ashes. Cover of the upper and lower tree layer was significantly negatively correlated with NMDS axis 1.

Cover of mosses and plots of the landscape type ancient moraines displayed a significant positive correlation with NMDS axis 2, while plots of the landscape type recent moraines were negatively correlated with this axis. In addition, NMDS axis 3 was most strongly positively correlated with height of adult ashes. Abiotic soil variables pH, CN and CEC, by the majority, did not display significant correlations with neither of the first three NMDS axes. Base saturation was the only variable that significantly correlated with NMDS axis 1.

### Crown Defoliation of Adult Ash Trees

**FIGURE 3:**
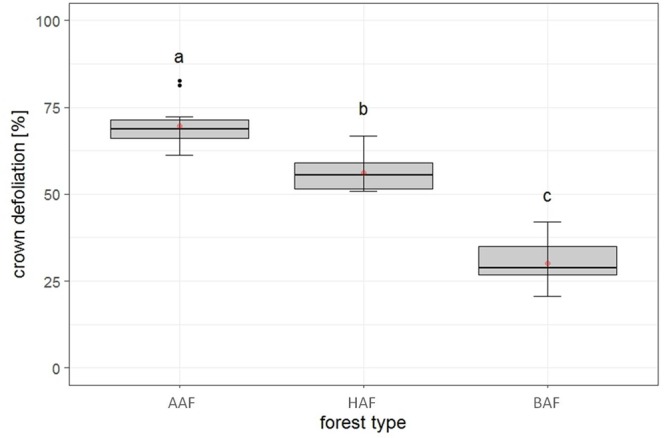
Plot level: Estimates of crown defoliation of adult ashes by forest type AAF, alder-ash forest; HAF, hornbeam-ash forest; BAF, beech-ash forest. Box plots give medians, quartiles, minimum, and maximum per forest type (*n* = 12 for AAF and HAF, *n* = 13 for BAF). Different letters indicate significant differences according to *post hoc* test. For statistical details see Table 2.

The first model tested for effects of several biotic and abiotic factors on mean crown defoliation of adult ashes on the plot level. After model selection, the optimal model was run with forest type, landscape type and total species richness, as predictors. Forest type was the only variable to display a significant effect on mean crown defoliation (Table 2). Crown defoliation was highest in the alder-ash forest, significantly lower in hornbeam-ash forest and significantly lowest in beech-ash forest (Figure 3). Total species richness slightly failed to be significant and landscape type did not have any significant effect on mean crown defoliation (Table 2).

**Table 2:** Results of the Analysis of Variance on the optimal linear mixed effects model explaining loss of leaves of adult ash trees on the plot level.

	MS	NumDF	DenDF	*F*	*P*	SigCode
**Forest type**	**4441.6**	2	27.2	**23.183**	**<0.001**	**^∗∗∗^**
Landscape type	558.1	1	16.75	2.9131	0.106
Species richness	776	1	24.5	4.0504	0.055

*Variables in bold show significant correlations. SigCode, significance code (0 “^∗∗∗^” 0.001 “^∗∗^” 0.01 “^∗^” 0.05 “.” 0.1 “ ” 1).*

**FIGURE 4:**
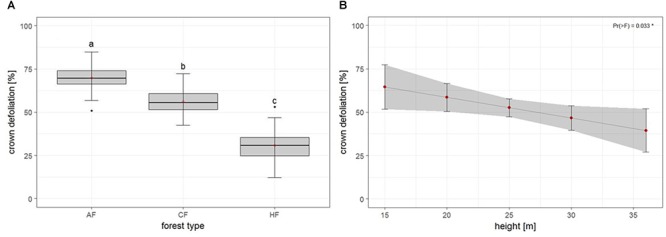
Individual level: Estimates of crown defoliation of adult ashes **(A)** by forest type and **(B)** in relation to tree height. AAF, alder-ash forest; HAF, hornbeam-ash forest; BAF, beech-ash forest. Box plots give medians, quartiles, minimum, and maximum per forest type (*n* = 12 for AAF and HAF, *n* = 13 for BAF). Different letters indicate significant differences according to *post hoc* test. For statistical details see Table 3.

The second model tested for effects of biotic and abiotic factors on crown defoliation of adult ash trees at the individual level. Again, forest type displayed a highly significant effect on crown defoliation (Figure 4A and Table 3), Crown defoliation of individual adult ashes was highest in the alder-ash forest, significantly lower in hornbeam-ash forest and significantly lowest in beech-ash forest (Figure 4A). In addition, crown defoliation significantly co-varied with the height of the individual ash tree: The model predicted larger trees to exhibit significantly less crown defoliation compared to smaller trees (Figure 4B), while diameter at breast height did not show such a significant effect on crown defoliation (Table 3).

**Table 3:** Results of the Analysis of Variance on the optimal linear mixed effects model explaining loss of leaves of adult ash trees on the individual tree level.

	MS	NumDF	DenDF	*F*	*P*	SigCode
**Forest type**	**8951.1**	**2**	**30.219**	**15.386**	**<0.001**	^∗∗∗^
Landscape type	998.1	1	28.538	1.716	0.201
Landscape type × forest type	206.6	2	29.836	0.355	0.704
**Height**	**2683**.8	**1**	**157.46**	**4.613**	**0.033**	^∗^
Diameter at breast height	143.1	1	148.74	0.246	0.621

*Variables in bold show significant correlations. SigCode, significance code (0 “^∗∗∗^” 0.001 “^∗∗^” 0.01 “^∗^” 0.05 “.” 0.1 “ ” 1).*

There was neither an effect of landscape type nor of landscape type × forest type interaction on crown defoliation (Table 3).

### Damage to Rejuvenation of Ash

**FIGURE 5:**
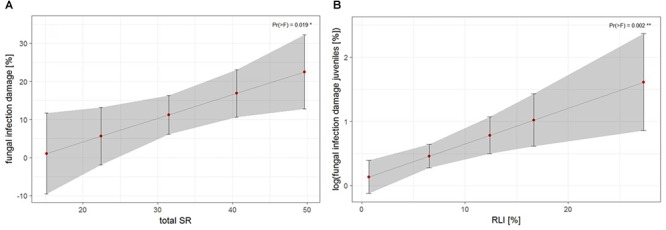
Estimates of fungal infection damage of juvenile ashes on the plot level, **(A)** based on *mean abundance of fungal infection* of juveniles in relation to total species richness (SR) and **(B)** log fungal infection based on *mean percental damage* of juveniles in relation to relative light intensity (RLI). For statistical details see Table 4.

The third approach examined the relationship of biotic and abiotic factors with infection damage of juvenile ashes per plot. For mean abundance of fungal infection, total species richness was the only significant predictor for infection damage after model selection (Table 4). Infection damage was positively correlated with total species richness, juvenile ashes in plots with a higher species richness were predicted to exhibit higher fungal damage (Figure 5A). For mean percental damage of juveniles, model selection suggested only RLI as a significant predictor (Table 4). Log infection of juveniles increased significantly with increasing light availability in the understorey (Figure 5B).

**Table 4:** Results of the Analysis of Variance on the optimal linear mixed effects model explaining damage to young ashes (a) based on mean abundance of fungal infection of juveniles and (b) based on mean percental damage of juveniles.

Variable	MS	NumDF	DenDF	*F*	*P*	SigCode
(a) *Abundance of fungal infection*
**Species richness**	**847.56**	**1**	**34.58**	**6.017**	**0.019**	**^∗^**
(b) *Percental damage of juveniles*
**RLI**	**3.144**	**1**	**35**	**10.698**	**0.002**	**^∗∗^**

*Variables in bold show significant correlations. SigCode, significance code (0 “^∗∗∗^” 0.001 “^∗∗^” 0.01 “^∗^” 0.05 “.” 0.1 “ ” 1).*

## Discussion

### Infection Damage to Ash Individuals

Based on data at the plot level as well as at the individual tree level, forest type was the strongest predictor for crown defoliation thereby confirming the first hypothesis. The models predict damage to be highest in the wet alder-ash forest (AAF), lower in the hornbeam-ash forest (HAF), and least in the beech-ash forest (BAF). This finding was supported by the NMDS as the variables for damage class and crown defoliation are strongly positively correlated with axis 1 and the forest types separate along this axis.

Such floristic gradients as encountered, in principal, may be due to several environmental factors. However, in the present study, strong evidence points toward soil moisture being the major influential factor. The investigated forest types line up perfectly along a soil moisture gradient (Härdtle, 1995). Although soil moisture was not measured directly in the field, the floristic gradient along the first NMDS axis can be interpreted as a gradient of increasing soil moisture: From beech-ash forests (BAF) with characteristic species as *Fagus sylvatica* L., *Melica uniflora* RETZ. and *Lamium galeobdolon* (L.) L. on the left side and with wetness indicators as *Mentha aquatica* L. and *Solanum dulcamara* L. more on the right side associated with alder-ash forests (AAF). Several authors stated that, on all sites, ashes may be subject to infection (Kowalski and Holdenrieder, 2008; NW-FVA, 2011; Lenz and Straßer, 2016). In other studies, in contrast, a more severe damage was observed on wet sites, which corresponds well with our present findings (Kenigsvalde et al., 2010; Schumacher, 2011; Vacek et al., 2015; Lenz and Straßer, 2016; Timmermann et al., 2017). There are several scenarios that might apply. Most prominently, this current finding applies because the fungus *H. fraxineus* thrives better in wet microclimatic conditions (Berger et al., 2010) as such conditions promote its spore production (Kirisitis and Cech, 2009; Timmermann et al., 2011, 2017; Thomas, 2016). In addition, one might argue that increasing levels of soil moisture also reflect situations where *F. excelsior* reaches the limits of its fundamental niche, which affects performance of the tree species and thus increases susceptibility to infection. However, in the present study, we included three forest types only; in future studies, it would thus be important to broaden the range of forest community types tested to increase the degree to which these results can be generalized.

While the extent of the infection damage to adult ash trees obviously differed between the three investigated forest types, such a significant trend could not be confirmed for juvenile ashes. The second hypothesis, therefore, has to be rejected. Forest type did not appear to be a significant predictor for infection damage to juvenile ashes. A possible explanation for this result could be that the general higher infection pressure close to the litter layer, compared to higher strata within stands (cf. Chandelier et al., 2014), alleviates the effect of site conditions. As an alternative, one might assume that abiotic covariables might exert a stronger effect on juveniles than on adult trees in explaining infection damage.

### Infection Damage and Abiotic Covariates

Several abiotic covariates as chemical soil parameters and understorey light conditions were suspected to potentially influence the extent of ash dieback. Firstly, we expected infection damage of adult ash trees and juvenile ashes to vary between the two landscape types ancient and recent moraines. However, the model predicted no significant differences for the landscape types neither for infection of the adult trees nor for fungal damage of the juveniles. Although there are principal differences between the predominant soil types of both landscapes, the subtypes can be ecologically relatively similar at the small scale (Härdtle, 1995). Basically, none of the chemical soil parameters quantified in this study displayed a significant effect for explaining the extent of the disease, thereby indicating that chemical soil parameters may not play a key role in influencing the extent of the disease. This is in line with several studies stating that ashes may become infected on all sites (Kowalski and Holdenrieder, 2008; NW-FVA, 2011; Lenz and Straßer, 2016). On the other hand, there is some evidence that ashes on soils in contact with groundwater (gleysols) may be more severely affected than on drier sites with stagnosols or luvisols (Berger et al., 2010; Kenigsvalde et al., 2010; Schumacher, 2011). Furthermore, these studies report high infection of *F. excelsior* at sites with organic soils. However, alder-ash forests at sites with organic soils were not considered in the present study because these ecosystems do not belong to the natural habitat of common ash and, furthermore, due to low species richness and the lack of endangered species they have not yet played an important role for nature protection (Schrautzer et al., 2007). However, for a more comprehensive ecological understanding of the role of site conditions for the development of the disease, the scope of habitat types should be broadened in future approaches.

In contrast, for juvenile ash individuals, we found a significant relationship between mean percental damage of juvenile individuals and understorey RLI in the final model. Although seedlings of *F. excelsior* can persist in shady conditions, they thrive better when more light is available (Wardle, 1959; Marigo et al., 2000). Apparently, this may coincide with a higher susceptibility at the individual level of juveniles. Admittedly, at this point, we are at the limits of interpretation: the extent as to which this finding is due to a causal relationship or a covarying outcome of forest community composition in the tree layer, needs to be addressed with experimental means in future approaches.

### Infection Damage and Biotic Covariates

Measures of size and vitality of ashes were studied on the individual tree level as biotic covariates, and species richness and abundance of ash on the plot level. Analyzing individual adult ash trees, height of the tree was found to be a strong significant predictor of crown defoliation, with larger trees showing less crown defoliation than smaller ones. Height of a tree can be seen as a measure for its age and vitality (Dobbertin, 2005). Our study suggests that older and more vigorous ash trees currently show less symptoms of the disease. Bengtsson et al. (2013) and Timmermann et al. (2017) found a similar relationship between the extent of infection damage and age or vitality of the ash tree in Sweden and in Norway, respectively. An explanation for this phenomenon could simply be that the larger the tree is in size, the longer it takes for the fungus to spread through the tree (Berger et al., 2010). Disease symptoms may appear later in large trees (Thomas, 2016) as the fungus takes more time spreading through a large than through a small tree and additionally more fungal damage is needed to harm a large tree individual. Further studies, accordingly, suggest that ash trees of all ages or sizes are lethally infected by the disease (Pautasso et al., 2013). Nevertheless, to date, the extent of damage is not the same for all ash trees (Lenz and Straßer, 2016) and has been found to differ between age groups (Kenigsvalde et al., 2010; Timmermann et al., 2017). In addition, more attention has to be paid not only to crown symptoms, but also to collar rots associated with the ash dieback (Enderle et al., 2017). However, it has to be considered that, in our study, the forest type with highest rate of infection, the wet alder-ash forest, exhibits also the smallest adult tree individuals. So, while in our data, there is a covariation between forest type and tree size, this might indicate a true interaction effect of both, site condition and growth potential of trees, on pattern of damage. Yet, this cannot be appropriately resolved with the observational data at hand.

Species richness is a non-significant predictor for crown defoliation of adult ashes on the plot level. However, in the NMDS, damage to adult ashes and species richness are significantly positively correlated with NMDS axis 1, and therefore indicate some covariation. Regarding the abundance of fungal damage occurrence on juvenile ashes, species richness is the only significant predictor: The model predicts juvenile ashes to show more fungal damage in more species-rich sites. Previous studies have found that a disease transmitted by pathogens has less severe effects in species-rich compared to species-poor ecological communities (Keesing et al., 2006; Haas et al., 2011). This has been found to apply both to generalist (Haas et al., 2011) and specialist fungal pathogens (Mitchell et al., 2002). Authors commonly mention the “dilution effect” (Keesing et al., 2006) as cause for the observed effect. At a higher level of species diversity, host density is reduced and this reduced host density is the underlying cause for reduced transmission and severity of the disease (Keesing et al., 2006). For the present data, however, it is more probable that species richness co-varies with infection damage on young ashes.

## Conclusion

In conclusion, the present data suggest a syndrome of factors, among those forest types (in particular those with a high species richness) associated with wet soil conditions, relatively low performance of tree individuals, building a relatively open canopy with thus high light availability in the understorey, to offer a suitable setting for the disease to develop. To identify whether all these factors only act in concert or more exclusively via some of its separate components, needs to be studied within a broader framework, ideally complemented by experimental planting trials, in the future.

## Data Availability

The datasets generated for this study are available on request to the corresponding author.

## Author Contributions

AE and JS conceived the ideas and designed the methodology. KH, L-MB, JR, and MB collected the data. KH and AE analyzed the data. AE, KH, and JS led the writing of the manuscript. All authors contributed critically to the drafts and gave final approval for the publication.

## Conflict of Interest Statement

The authors declare that the research was conducted in the absence of any commercial or financial relationships that could be construed as a potential conflict of interest.
